# Does adding risk-trends to survival models improve in-hospital mortality predictions? A cohort study

**DOI:** 10.1186/1472-6963-11-171

**Published:** 2011-07-21

**Authors:** Jenna Wong, Monica Taljaard, Alan J Forster, Carl van Walraven

**Affiliations:** 1 Clinical Epidemiology Program, Ottawa Hospital Research Institute, 1053 Carling Avenue, Ottawa, K1Y 4E9, Canada; 2 Department of Medicine, University of Ottawa, 1053 Carling Avenue, Ottawa, K1Y 4E9, Canada

## Abstract

**Background:**

Clinicians informally assess changes in patients' status over time to prognosticate their outcomes. The incorporation of trends in patient status into regression models could improve their ability to predict outcomes. In this study, we used a unique approach to measure trends in patient hospital death risk and determined whether the incorporation of these trend measures into a survival model improved the accuracy of its risk predictions.

**Methods:**

We included all adult inpatient hospitalizations between 1 April 2004 and 31 March 2009 at our institution. We used the daily mortality risk scores from an existing time-dependent survival model to create five trend indicators: absolute and relative percent change in the risk score from the previous day; absolute and relative percent change in the risk score from the start of the trend; and number of days with a trend in the risk score. In the derivation set, we determined which trend indicators were associated with time to death in hospital, independent of the existing covariates. In the validation set, we compared the predictive performance of the existing model with and without the trend indicators.

**Results:**

Three trend indicators were independently associated with time to hospital mortality: the absolute change in the risk score from the previous day; the absolute change in the risk score from the start of the trend; and the number of consecutive days with a trend in the risk score. However, adding these trend indicators to the existing model resulted in only small improvements in model discrimination and calibration.

**Conclusions:**

We produced several indicators of trend in patient risk that were significantly associated with time to hospital death independent of the model used to create them. In other survival models, our approach of incorporating risk trends could be explored to improve their performance without the collection of additional data.

## Background

Many physicians informally prognosticate patients by determining changes in their health status over time. Physicians assess whether patients are getting better, getting worse, or staying the same by comparing their current health state to that quantified from previous assessments. Conclusions made from such assessments are essential for gauging a patient's present status and predicting future outcomes.

Findings from several studies suggest that trends (i.e. changes over time) in prognostic factors may play an important role in predicting patient outcomes. In prostate cancer patients, changes in health-related quality of life measurements were associated with mortality [1] and other clinical outcomes [2]. In chronic heart failure patients, relative changes in the level of N-terminal pro-brain natriuretic peptide [3] and changes in peak oxygen consumption [4] were found to be associated with the risk of death. In cardiac surgery patients, even small changes in serum creatinine after surgery were found to predict subsequent death independent of other established perioperative risk factors [5]. In each of these studies, changes over time were measured *prior *to the analytical baseline and were not measured during the observation period. Continual measurement of changes in prognostic markers throughout the observation period could further improve risk prediction.

We recently derived and internally validated a time-dependent survival model for hospital death that could predict a patient's daily mortality risk via estimation of the hazard of death on each day [6]. The model expanded on that of Escobar *et al*. [7] by using Cox (instead of logistic) regression methods and including time-dependent covariates. Our model had good discrimination (concordance probability = 0.879) and calibration (close agreement between the observed and expected number of deaths in all risk strata over all admission days). Our model's commendable predictive performance was largely due to the inclusion of time-dependent covariates, whose values could be updated on a daily basis [unpublished data, Wong *et al*.]. However, our model used only the most recent covariate values to predict a patient's daily risk of hospital death and did not consider trends over time in a patient's covariate values or risk of death prior to that day.

In this study, we incorporated indicators of trend in patient risk into our time-dependent model. We first calculated daily risk scores (summary indices of risk based on a patient's daily covariate values) from our existing model and used these to generate several indicators of trend in patient risk. We then re-estimated our survival model including these trend indicators *in addition *to the original model covariates. We did this to determine whether prior changes in a patient's risk of hospital death were predictive of daily death risk independent of the original model covariates. Finally, we compared the predictive performance of the existing model with and without the trend indicators to determine if these markers significantly improved the model's ability to predict daily risk of hospital mortality.

## Methods

### Study design and setting

This was a cohort study including all inpatient hospitalizations that occurred between 1 April 2004 and 31 March 2009 at The Ottawa Hospital (TOH). TOH is a tertiary-care teaching hospital in Ottawa, Canada that consists of two inpatient campuses, operates within a publicly funded health care system, and serves a population of approximately 1.5 million people in Ottawa and Eastern Ontario. All data in this study came from a large repository of administrative and laboratory data originating from the hospital's major operational systems. We derived all models on a randomly selected 66% of hospitalizations and assessed their performance on the remaining admissions. The unit of analysis in the study was the hospitalization. This study was approved by the TOH Research Ethics Board.

### Study cohort

We used the same set of admissions as that used previously to derive and internally validate our time-dependent survival model [6]. This cohort included all inpatient admissions at our hospital during the study period except those where the patient was younger than 15 years of age, transferred to or from another acute care hospital, or hospitalized for obstetrical reasons. Hospitalizations of patients transferred between TOH campuses were linked and considered a single admission.

### Time-dependent survival model for hospital mortality

The time-dependent model for hospital mortality was derived using Cox regression methods [6]. The model included three time-independent covariates whose values remained constant over the hospitalization: patient age^2 ^(expressed as a restricted cubic spline); admission type (emergent surgical and non-surgical, elective surgical and non-surgical); and the Elixhauser score [8] to summarize the patient's comorbidities at admission.

The model also included four time-dependent covariates whose values could be updated on daily basis: intensive care unit (ICU) status - a binary variable whose value was '1' for patients in the ICU at the beginning of the day ('0' otherwise); alternative level of care (ALC) status - a binary variable whose value was '1' for patients awaiting placement in a long-term care facility and no longer receiving active medical care ('0' otherwise); the *P*rocedure *I*ndependent *M*ortality *R*isk score [unpublished data, van Walraven *et al*.] - a continuous variable summarizing the performance of important therapeutic procedures independently associated with hospital death risk; and the number of days elapsed since the last PIMR procedure (expressed as x^-0.5^). The daily value of the PIMR score was equal to the score of the most recently performed PIMR procedure ('0' if no PIMR procedure had been performed previously, or the sum of the individual scores if more than one PIMR procedure was performed on a given day).

Finally, the model included a number of interaction terms between covariates and between specific time-independent variables and a logarithmic transformation of time. Details of this model are described elsewhere [6].

### Markers of daily death risk from the time-dependent model

The time-dependent survival model could produce two different, but related estimates of daily death risk. First, the model could estimate a patient's hazard of death on each hospitalization day (Appendix). The hazard on a given day represents the instantaneous rate of death on that day and can be interpreted as a patient's risk of death on that day conditional upon his or her survival up to that day.

Second, the model could produce a daily "risk score" by summing the product of each covariate value and its regression coefficient from the model (i.e. Σβx) on each hospitalization day. The risk score is related to the hazard because the latter is calculated by multiplying the exponential of the risk score (i.e. *e*^Σβx^) by the baseline hazard estimate (i.e. the hazard for a patient with all covariate values set to 0 or the reference value).

In this study, we used the daily risk score instead of the hazard to measure changes over time in a patient's daily death risk. This is because changes in the hazard over time are due to changes in a patient's covariate values *as well as *fluctuations in the baseline hazard over time. In contrast, changes in the daily risk score are directly proportional to changes in a patient's covariate values and therefore more closely reflect changes in a patient's condition.

### Indicators of trend in daily death risk

We created five time-dependent variables to quantify the direction, degree, and duration of change in a patient's risk score prior to each hospitalization day. These variables (which we refer to as "trend indicators") were: 1) the absolute change in the risk score from the previous day; 2) the relative percent change in the risk score from the previous day; 3) the absolute change in the risk score from the start of the trend; 4) the relative percent change in the risk score from the start of the trend; and 5) the number of consecutive days with a trend in the risk score. We defined a trend as a period of time over which the daily risk score increased or decreased over consecutive days. We expressed all of the trend indicators as continuous variables, using a positive or negative number to indicate an increasing or decreasing risk score, respectively. If there was no change in the risk score or no previous risk score for comparison (i.e. on the first day of the hospitalization), the value of all trend indicators was set to 0.

### Incorporation of trend indicators into the existing time-dependent model

We first added the individual trend indicators (expressed as time-dependent covariates) separately to the existing time-dependent survival model to determine whether each indicator was significantly associated with time to hospital death (*independent *of the covariates in the existing model) and whether the relative or absolute change was more informative. We used the likelihood ratio test to determine the statistical significance of each trend indicator and compared the prognostic value of the trend indicators using the value of Akaike's Information Criterion (AIC) for each model.

Next, we jointly added only those trend indicators that were individually significant (based on the likelihood ratio test) to the existing model. If the relative and absolute change in the risk score from the previous day or from the start of the trend were individually significant, we only added the trend indicator with the greatest prognostic value (i.e. that which produced the model with the lowest AIC when added separately to the existing model) to avoid multi-collinearity. We then used the methods of Sauerbrei and Royston [9] to identify the best second-degree fractional polynomial transformation for each trend indicator in the joint model and removed any trend indicators that were no longer significant at α = 0.05.

We derived all models using a randomly selected 66% of hospitalizations (the derivation set). For all analyses, we used the PHREG procedure in SAS, Version 9.2 (Cary, NC).

### Performance of the existing model with and without the trend indicators

In the remaining third of admissions (the validation set), we compared the predictive performance of the existing model with and without the trend indicators using four different methods.

First, we measured the discrimination of each model by calculating the concordance probability (also known as the area under the ROC curve) with 95% confidence intervals (CIs) [10]. The concordance probability represents the proportion of all informative pairs of patients where the patient with the lower model-predicted risk of death survives longer.

Second, we measured the calibration of each model by dividing admissions into risk deciles on each admission day and calculating the number of observed and expected deaths within each risk decile on each day. We calculated the number of expected deaths by summing the hazard on that day of all patients within each decile. We then summed the number of observed and expected deaths within each risk decile across all admission days to give the final estimate. In order to directly compare the calibration of the two models, we used the *same *risk decile groupings (based on the daily risk score from the new model with the trend indicators). To test for a significant difference between the number of observed and expected events within each decile, we calculated the p-value associated with the standardized z-statistic [11].

Lastly, we calculated the Integrated Discrimination Improvement (IDI) and the Net Reclassification Improvement (NRI) [12] to quantify the improvement in predictive performance attained by adding the trend covariates to the existing model. The IDI represents the difference in discrimination slopes between the new and existing model (where the discrimination slope is the mean predicted risk of death among patients who died minus the mean predicted risk of death among patients who survived). The IDI can also be interpreted as the change in average sensitivity minus the change in average (1-specificity). An IDI above zero indicates improved discrimination with the new model. We calculated the IDI with 95% CIs [12] on *each *admission day up to day 32 (the 95^th ^percentile of observed length of stay) using patients' hazard estimate and vital status *for that day*. We calculated the IDI on each admission day because patients' covariate values and hazard of death could change daily.

The NRI quantifies the amount of correct reclassification (i.e. upwards for events and downwards for non-events) when comparing the predicted risk of death from the new versus the existing model. Specifically, the NRI is calculated as the proportion of correct minus incorrect reclassifications among events (i.e. patients who died) plus the proportion of correct minus incorrect reclassifications among non-events (i.e. patients who were discharged alive). An NRI above zero indicates improved risk prediction with the new model. Because no established risk categories for hospital mortality exist, we calculated a category-less NRI, where upward reclassification was defined as a higher predicted risk of death from the new model and downward reclassification was defined as a lower predicted risk of death from the new model [13]. As with the IDI, we calculated the NRI with 95% CIs [12] on each of the first 32 admission days using patients' hazard estimate and vital status for that day.

## Results

A total of 159 787 hospitalizations were used previously to derive and internally validate our existing time-dependent survival model [6]. Characteristics of the derivation and validation sets were very similar (Table 1). Most commonly, hospitalizations were emergent and non-surgical and involved elderly patients (median age 61). At admission, most patients had few or no comorbidities (median Elixhauser score of 0), a fairly low acuity of illness (median LAPS of 4 in the validation set), and a low estimated hazard of death (median hazard of 0.00008, as predicted by the existing time-dependent model). PIMR procedures were performed during 28% of hospitalizations, most of which were low-risk procedures (median PIMR score of 1). Only 3% of hospitalizations had a PIMR procedure performed on more than one day. Few patients were admitted to the ICU (5%) or awaited placement (4%) during the hospitalization. Most admissions were fairly short (median length of stay of 5 days). Over the study period, 5% of admissions ended in death.

**Table 1:** Characteristics of admissions included in the study

Characteristic	Derivation	Validation
Patients/Hospitalizations, n*	77294/106522	44300/53265
Deaths in-hospital, n (%)	5407 (5.1)	2640 (5.0)
Length of admission in days, median (IQR*)	5 (2-9)	5 (2-9)
Male, n (%)	55295 (51.9)	27807 (52.2)
Age at admission, median (IQR)	61 (48-75)	61 (48-74)
Admission type, n (%)		
Emergent non-surgical	49862 (46.8)	24982 (46.9)
Emergent surgical	22534 (21.2)	11187 (21.0)
Elective non-surgical	14184 (13.3)	6970 (13.1)
Elective surgical	19942 (18.7)	10126 (19.0)
Elixhauser score, median (IQR)	0 (0-6)	0 (0-6)
LAPS* at admission, median (IQR)	5 (0-38)	4 (0-38)
Hazard of death at admission^†^, median (IQR)	0.0008 (0.0002- 0.0040)	0.0008 (0.0002- 0.0039)
At least 1 admission to the intensive care unit, n (%)	5433 (5.1)	2654 (5.0)
Change from active care to alternative level of care, n (%)	4830 (4.5)	2363 (4.4)
At least 1 PIMR* procedure, n (%)	29791 (28.0)	14923 (28.0)
PIMR score on day of procedure^‡^, median (IQR)	1 (-4 - 2)	1 (-4 - 2)

*n = number; IQR = interquartile range; LAPS = Laboratory-based Acute Physiology Score; PIMR = Procedure Independent Mortality Risk

^†^as predicted by the existing time-dependent survival model

^‡^among admissions where at least 1 PIMR procedure was performed. For admissions where PIMR procedures were performed on more than one day (3% of all admissions), we used the PIMR score on the first procedure day to calculate the median (IQR) score. If more than one PIMR procedure was performed on the procedure day, the scores of the individual procedures were summed. A negative PIMR score indicates a procedure associated with a decreased risk of hospital death.

**Figure 1:**
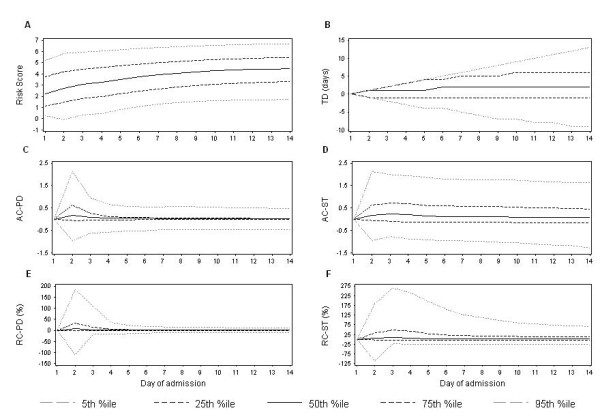
**Distribution of the risk score from the existing time-dependent survival model and the trend indicators over time**. The graphs above show the value of the risk score from the existing time-dependent survival model (Figure 1A) and the five trend indicators (Figure 1B-1F) at the 5^th^, 25^th^, 50^th^, 75^th^, and 95^th ^percentile of observed values on each of the first 14 days of admission. The value of each trend indicator is positive or negative for an increase or decrease in the risk score, respectively. TD = number of consecutive days with a trend in the risk score; AC-PD = absolute change in the risk score from the previous day; AC-ST = absolute change in the risk score from the start of the trend; RC-PD = relative percent change in the risk score from the previous day; RC-ST = relative percent change in the risk score from the start of the trend.

The median value of the risk score from the existing model increased over time, while the variability of the risk score remained constant (Figure 1A). The median value of each of the trend indicators was fairly stable over time (Figure 1B-1F). Variability of the absolute change in the risk score from the start of the trend remained stable over time (Figure 1D). In contrast, variability increased over time for the number of consecutive days with a trend in the risk score (Figure 1B) and decreased over time for the remaining trend indicators. Compared to the risk score from the previous day, the risk score on days 2, 3, and 4 increased in 70%, 66%, and 65% of patients, respectively, and decreased in 30%, 34% and 35% of patients, respectively. The risk score did not stay the same for any patients because the existing model included interaction terms with time (which altered the risk score even if the covariate values remained the same).

When we added each trend indicator separately to the existing model, three trend indicators were independently and strongly significant: the absolute change in the risk score from the previous day; the absolute change in the risk score from the start of the trend; and the number of consecutive days with a trend in the risk score (Table 2). Of these three covariates, the absolute change from the start of trend had the greatest prognostic value because it produced the model with the lowest AIC (Table 2).

**Table 2:** Significance of each trend indicator when added separately to the existing time-dependent model

Trend indicator added to the existing model^‡^	-2 log likelihood (46389.92^†^)	*p*-value*	AIC** (46445.92^†^)
Absolute change in the risk score from the previous day	46340.45	< .0001	46398.45
Absolute change in the risk score from the start of the trend	46303.56	< .0001	46361.56
Relative change in the risk score from the previous day	46389.70	0.6386	46447.70
Relative change in the risk score from the start of the trend	46389.87	0.8361	46447.87
Number of consecutive days with a trend in the risk score	46377.89	0.0005	46435.89

^†^for the existing time-dependent model with no trend indicators

*p-value from the likelihood ratio test comparing the existing time-dependent model with and without the trend indicator

**Akaike's Information Criterion

**^‡^**For all trend indicators, the value was expressed as a positive or negative number for an increase or decrease in the risk score, respectively. We defined a trend as a period of time over which the risk score *consistently *increased or decreased. If there was no change in the risk score or no previous risk score for comparison (i.e. on the first day of the hospitalization), the value of all trend indicators was set to 0.

These three trend indicators were still highly significant (*p *< .0001) when added jointly to the existing model using the following transformations: X^3 ^+ X^3^*log(X) for the absolute change in the risk score from the previous day; X^0.5 ^+ X^0.5^*log(X) for the absolute change in the risk score from the start of the trend; and X^-2 ^+ X^-2^*log(X) for the number of consecutive days with a trend in the risk score (we first shifted all original values up by the minimum observed value to ensure the values were greater than zero). Moreover, all of the original covariates in the existing model remained statistically significant in the presence of these trend indicators.

**Figure 2:**
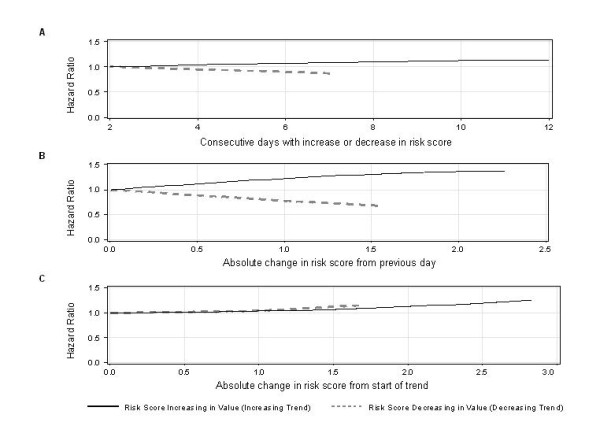
**Effect of the trend indicators on the hazard of death in hospital**. The graphs above show the multiplicative effect of different values of each trend indicator on the hazard of death (compared to the hazard for a patient with the minimum absolute value of the trend indicator). To create the graphs, we held the value of all covariates constant (except the trend indicator of interest) and calculated the risk score when the value of the trend indicator of interest was allowed to vary from a minimum absolute value up to the 95^th ^percentile of observed values for an increasing trend (black solid line) or down to the 5^th ^percentile of observed values for a decreasing trend (grey dotted line). We calculated the hazard ratios by exponentiating (i.e. *e*^x^) the difference between each risk score and the risk score for the minimum absolute value of the trend indicator.

In the final model, the trend indicators notably changed the predicted risk of death in hospital. The adjusted hazard ratio increased as the number of days with an increasing trend in the risk score increased (Figure 2A, solid line), the absolute change in the risk score from the previous day increased (Figure 2B, solid line), and the absolute change in the risk score from the start of the trend increased (Figure 2C, solid line). The adjusted hazard ratio decreased as the number of days with a decreasing trend in the risk score increased (Figure 2A, dotted line) and as the absolute change in the risk score from the previous day decreased (Figure 2B, dotted line). Contrary to our expectations, the hazard ratio increased for larger *decreases *in the risk score from the start of the trend (Figure 2C, dotted line).

However, the addition of the trend indicators to the existing model produced only a marginal, non-significant improvement in the concordance probability. The concordance probability of the existing model was 0.8790 (95% CI 0.8718-0.8861) compared to 0.8801 (0.8730-0.8873) for the new model, representing an absolute increase of only 0.11%.

The calibration of both models was excellent. Within each risk decile, the number of deaths predicted by each model was not significantly different from the number of observed deaths (Table 3). However, the predictions from the model that incorporated trend indicators were not noticeably better. This model predicted slightly closer to the number of observed deaths in six risk deciles, while the existing model (without trend indicators) predicted slightly closer in the other four deciles (Table 3). Overall, the model incorporating trend indicators predicted closer to the total number of deaths, but only by 12 deaths compared to the number of deaths predicted by the existing model (Table 3 last row).

**Table 3:** Calibration of the existing time-dependent model with and without the trend indicators

		Existing model with trend indicators			Existing model without trend indicators		
		
Risk Decile	# observed deaths	# expected deaths	z-score	*p*	# expected deaths	z-score	*p*
1	5	4.31	0.3298	0.7415	5.37	0.1609	0.8722
2	9	12.75	1.0498	0.2938	13.68	1.2664	0.2054
3	21	24.74	0.7527	0.4517	26.03	0.9853	0.3245
4	36	43.45	1.1298	0.2586	44.59	1.2861	0.1984
5	54	68.90	1.7952	0.0726	69.50	1.8597	0.0629
6	103	108.72	0.5482	0.5836	109.97	0.6646	0.5063
7	174	171.65	0.1796	0.8575	173.49	0.0386	0.9692
8	260	285.35	1.5008	0.1334	285.02	1.4821	0.1383
9	453	474.99	1.0090	0.3130	478.93	1.1850	0.2360
10	1525	1496.72	0.7309	0.4649	1497.23	0.7177	0.4729
**Total**	**2640**	**2691.59**	**0.9943**	**0.3201**	**2703.82**	**1.2274**	**0.2197**

We divided the validation admissions into risk deciles on each admission day (based on the patient's risk score for that day from the model *with *the trend indicators), determined the number of observed and expected deaths from each model within each risk decile on each day (where the daily number of expected deaths was equal to the sum of the daily hazard of all patients within each decile), and finally summed the number of observed and expected deaths within each risk decile *across all admission days *(shown above). We tested for a significant difference between the observed and expected number of deaths within each decile by calculating the *p*-value associated with standardized *z*-statistic, where *z *= (observed-expected)/(√expected).

**Figure 3:**
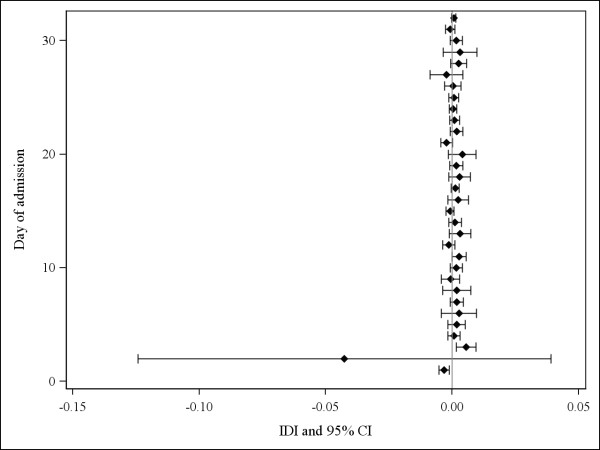
**Integrated discrimination improvement (IDI) with 95% confidence intervals (CI) comparing the existing model with and without the trend indicators**. On each of the first 32 admission days (the 95^th ^percentile of observed length of stay), we calculated the IDI with 95% CIs [12] using patients' estimated hazard of death from each model and vital status *for that day*. An IDI above 0 suggests that the addition of the trend indicators to the existing model improved overall model discrimination.

The IDI suggested that the addition of the trend indicators did not significantly improve the overall discrimination of the existing model. The IDI was not statistically significant on 29 out of 32 days (Figure 3). On days 3 and 11, the IDI was 0.006 and 0.003, respectively, and was statistically significant on both days (suggesting improvement in overall discrimination). However, on day 1, the IDI was below zero (-0.003) and statistically significant (suggesting *worse *overall discrimination). On day 2, the IDI was particularly negative (-0.043), but not statistically significant.

**Figure 4:**
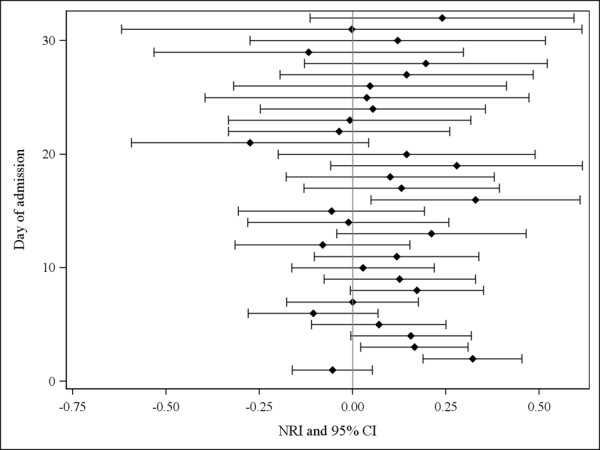
**Net reclassification improvement (NRI) with 95% confidence intervals (CIs) comparing the existing model with and without the trend indicators**. On each of the first 32 admission days (the 95^th ^percentile of observed length of stay), we calculated the NRI with 95% CIs [12] using patients' estimated hazard of death from each model and vital status *for that day*. An NRI above 0 indicates improved risk prediction with the new model containing the trend indicators.

The NRI also suggested that the addition of the trend indicators did not significantly improve the daily performance of the existing model. The NRI was not statistically significant on 29 days (Figure 4). On days 2, 3, and 16, however, the NRI was significantly above zero, with a value of 0.32, 0.16, and 0.33, respectively (Figure 4).

## Discussion

In this study, we determined if changes over time in a patient's health status were predictive of a patient's daily risk of hospital death *independent *of his or her current health status. To do this, we first used an existing time-dependent survival model for hospital mortality to produce summary indices of hospital death risk (i.e. risk scores) on each day of a patient's hospitalization. We then used these risk scores to generate indicators of trend (change over time) in a patient's risk of hospital death. Finally, we added these trend indicators to the existing survival model and determined whether their inclusion improved the model's predictive ability.

We found that three trend indicators (the absolute change in the risk score from the previous day; the absolute change in the risk score from the start of the trend; and the number of consecutive days with a trend in the risk score) were significantly and independently associated with the risk of hospital mortality. Moreover, when these trend indicators were added to the existing model, the original covariates remained statistically significant. However, we found that adding these trend indicators to the existing survival model did not noticeably improve its predictive performance.

We believe that the approach we used to measure changes over time in patient risk is unique and efficient. By simply using the daily risk scores produced by the existing time-dependent survival model, we were able to create a number of statistically significant trend indicators without having to collect additional patient data. Further studies should determine whether our approach is generalizable to other survival models that include time-dependent covariates.

The effect of larger absolute *decreases *in the risk score from the start of the trend was noteworthy (Figure 2C, dotted line). The model predicted a higher hazard of death for larger absolute *decreases *in the risk score over time. This suggests that patients with larger decreases in their risk score over time had worse outcomes than those with smaller decreases in their risk score. This observation could be due to sicker patients having higher risk scores to begin with and therefore experiencing larger decreases in their risk score over time. In contrast, relatively healthier patients may experience only small decreases in their risk score over time because their risk score is lower to begin with.

Several reasons could explain why the trend indicators were highly statistically significant (*p *< .0001) but did not notably improve the predictive performance of the existing model. Since the performance of the existing model was already very good, we needed new covariates with a very large and independent association with hospital mortality in order to produce noticeable improvements in the model's performance [12]. However, the magnitude of association between the trend indicators and the risk of hospital death was probably only moderate or even small since most hospitalizations were short (median stay of five days) and involved relatively healthy patients, thereby making it unlikely that the risk score would change drastically over most hospitalizations. Moreover, the trend indicators were likely highly statistically significant because of our large study size, which can make moderate or even small associations statistically significant.

While our approach of using model-generated risk scores to measure trends in patient health status is efficient and relatively simple, it is computationally intensive and may not appeal to those who find it too challenging or laborious. For example, even the calculation of the risk score itself could be challenging in cases where the existing model contains a large number of predictors and/or interaction terms. Another disadvantage of our approach is that it cannot reliably measure trends at the very start of the observation period (since there are no previous risk scores for comparison). On day 1, because there were no previous risk scores available for all admissions, we set the value of the trend indicators to zero (assuming neither an improving nor deteriorating health status). This may have been inaccurate for some patients and may have adversely affected the model fit early on, as suggested by the negative value of the IDI on days 1 and 2 (Figure 3). Therefore, we suggest that models including trend indicators be used for risk prediction only once patients have been observed long enough to measure a trend in their risk scores. In our case, just a few days may be sufficient since the value of the IDI was positive and statistically significant on day 3 (Figure 3).

## Conclusions

In this study, we demonstrate a unique and efficient approach to measure trends in patient risk over time. We used risk scores calculated on a daily basis from an existing time-dependent survival model for hospital mortality to create several trend indicator variables. When added to the existing model, these trend indicators were significantly associated with the risk of hospital death independent of the original variables in the existing model. However, the *clinical *significance of these trend indicators was minimal because they did not noticeably improve the predictive performance of the existing model. Future research should determine whether the approach we present in this study is generalizable to other time-dependent survival models and can significantly improve the performance of survival models with much longer time horizons where larger changes in patient risk are more likely.

## List of Abbreviations

AIC: Akaike's Information Criterion; ALC: Alternative Level of Care; CI: Confidence Interval; IDI: Integrated Discrimination Improvement; ICU: Intensive Care Unit; NRI: Net Reclassification Improvement; PIMR: Procedure Independent Mortality Risk; ROC: Receiver Operating Curve; TOH: The Ottawa Hospital.

## Competing interests

The authors declare that they have no competing interests.

## Authors' contributions

JW was involved in the study design, conducted the data analysis, interpreted the data, and led the drafting of the manuscript. CvW and MT participated in the study design, helped with the interpretation of the data, and reviewed the manuscript for its clinical and statistical content. AJF also participated in the study design and reviewed the manuscript for its clinical content. All authors have read and approved the final manuscript.

## Appendix: Calculation of the daily hazard of death from a time-dependent Cox model

The hazard of death at time *t *represents the instantaneous rate of death at *t *and can be interpreted as the risk of death at *t *conditional upon survival to *t*. The hazard of death can be estimated from a Cox regression model using the formula:

where *h*(*t*) is the estimated hazard of death on day *t*, Σ*βx *is the "risk score" (i.e. the sum of the product of each covariate value and its parameter estimate), and *h_0_*(*t*) is the estimated baseline hazard of death on day *t*.

To estimate a patient's hazard of death on each hospitalization day with our time-dependent Cox model, we used SAS to obtain daily estimates of the baseline hazard function and then multiplied the baseline hazard estimate for each day by the patient's "risk score" from the model for that day. Note that a patient's risk score could change over the hospitalization because the model included time-dependent covariates.

## Pre-publication history

The pre-publication history for this paper can be accessed here:

http://www.biomedcentral.com/1472-6963/11/171/prepub
